# Risk factors influencing survival of acellular porcine corneal stroma in infectious keratitis: a prospective clinical study

**DOI:** 10.1186/s12967-019-02192-z

**Published:** 2019-12-30

**Authors:** Saiqun Li, Meng Li, Li Gu, Lulu Peng, Yuqing Deng, Jing Zhong, Bowen Wang, Qian Wang, Yichen Xiao, Jin Yuan

**Affiliations:** State Key Laboratory of Ophthalmology, Zhongshan Ophthalmic Center, Sun Yat-sen University, Guangzhou, 510060 China

**Keywords:** Therapeutic lamellar keratoplasty, Infectious keratitis, Acellular porcine corneal stroma

## Abstract

**Background:**

A worldwide lack of donor corneas demands the bioengineered corneas be developed as an alternative. The primary objective of the current study was to evaluate the efficacy of acellular porcine corneal stroma (APCS) transplantation in various types of infectious keratitis and identify risk factors that may increase APCS graft failure.

**Methods:**

In this prospective interventional study, 39 patients with progressive infectious keratitis underwent therapeutic lamellar keratoplasty using APCS and were followed up for 12 months. Data collected for analysis included preoperative characteristics, visual acuity, graft survival and complications. Graft survival was evaluated by the Kaplan–Meier method and compared with the log-rank test.

**Results:**

The percentage of eyes that had a visual acuity of 20/40 or better increased from 10.3% preoperatively to 51.2% at 12 months postoperatively. Twelve patients (30.8%) experienced graft failure within the follow-up period. The primary reasons given for graft failure was noninfectious graft melting (n = 5), and the other causes included recurrence of primary infection (n = 4) and extensive graft neovascularization (n = 3). No graft rejection was observed during the follow-up period. A higher relative risk (RR) of graft failure was associated with herpetic keratitis (RR = 8.0, P = 0.046) and graft size larger than 8 mm (RR = 6.5, P < 0.001).

**Conclusions:**

APCS transplantation is an alternative treatment option for eyes with medically unresponsive infectious keratitis. Despite the efficacy of therapeutic lamellar keratoplasty with APCS, to achieve a good prognosis, restriction of surgical indications, careful selection of patients and postoperative management must be emphasized.

*Trial registration* Prospective Study of Deep Anterior Lamellar Keratoplasty Using Acellular Porcine Cornea, NCT03105466. Registered 31 August 2016, ClinicalTrails.gov

## Background

Infective keratitis is considered a major global cause of visual impairment [1–3]. In severe and progressive infections that are nonresponsive to medical treatment, therapeutic keratoplasty serves as a last resort to remove the infective focus or restore globe integrity. Therapeutic keratoplasty, including penetrating keratoplasty (PKP) and anterior lamellar keratoplasty (ALK), contributes to a significant proportion of corneal transplantations performed in the developing world, especially in Asia [4, 5]. Although keratoplasty works well in infective keratitis, the shortage of donor corneas is the major limiting factor for the performance of corneal transplantation in many countries, which leads to the development of biomimetic and implantable human corneal substitutes [6]. Many methods of stromal replacements have been proposed to date [7–9]. Recently, biosynthetic collagen hydrogel and acellular porcine corneal stroma (APCS) have been tested in a very limited number of human clinical trials with promising results [10, 11].

APCS is processed from fresh porcine corneas by removing nuclear and cellular materials [12]. The approved indication for APCS is the treatment of infectious keratitis. However, the effectiveness of APCS has so far been reported only in fungal keratitis and herpes simplex keratitis (HSK) [10, 11]. Therefore, to provide better guidance for clinical practice, we conducted this study to evaluate the efficacy of APCS keratoplasty in progressive infectious keratitis caused by a wide variety of pathogens, such as fungi, bacteria, viruses or *Acanthamoeba*. In addition, another purpose of the present study was to identify some of the factors that may increase the therapeutic failure rate of APCS transplantation so that future patients with a high risk of graft failure may be excluded and avoidance measures may be taken.

## Methods

### Patient population

Thirty-nine patients (39 eyes) undergoing APCS transplantation in Zhongshan Ophthalmic Center, Sun Yat-Sen University to treat medically uncontrolled infectious keratitis with a bacterial, fungal, *Acanthamoeba* or viral etiology were consecutively enrolled in this prospective cohort study. Infectious keratitis was defined as medically uncontrolled if the disease had significantly progressed after a 2-week of maximum antimicrobial medication or if the cornea was at risk of perforation. All participants completed 12 months of postoperative follow-up. The diagnosis of nonviral infectious keratitis was achieved by smear, microbial culture, confocal microscopy and pathologic examination. The diagnosis of HSK was based on multiple episodes of recurrence and clinical manifestations (typical epithelial dendritic lesions and stromal abnormalities). The exclusion criteria for APCS transplantation included an infiltration size larger than 9 mm and signs associated with infection that affected the corneal endothelium and anterior chamber. Hypopyon was not an absolute contraindication for ALK as long as it was due to severe intraocular sterile inflammation. Noninfectious hypopyon was distinguished from infectious hypopyon based on clinical appearance. Compared to infectious hypopyon, a noninfectious hypopyon is generally relatively more likely to shift with a change in head position. However, there is substantial overlap between these two entities. Moreover, if the lesions were noted to not extended to the level of Descemet’s membrane (DM), the hypopyon was presumed to be noninfectious. Nonetheless, the early intraocular spread of infection could not be fully excluded. It is even especially difficult to distinguish noninfectious hypopyon from infectious hypopyon in cases with a deep lesion. In light of these considerations, we prudently performed lamellar keratoplasty in cases with hypopyon.

### Corneal stroma substitutes

The APCS graft used in the current study was prepared by AiNear Corneal Engineering Corporation (Shenzhen, China) as previously reported [13]. After it was enucleated from a quarantined pigs immediately after its death, each fresh eyeball was thoroughly washed in sterile phosphate-buffered saline (PBS). Whole corneas with 2 mm of sclera were cut from the eyeballs using a 16-mm trephine and immersed into ultrapure water for 12 h to allow swelling. The corneoscleral tissue was then agitated for three times of 30 min in 2M NaCl followed by 30 min in ultrapure water. Next, the corneas were washed in 0.2% Triton X-100 for 6 h and then thoroughly washed in PBS to remove any remnant of Triton X-100 from the tissue. After it was dehydrated in glycerol until it reached a normal natural corneal thickness, the APCS was trimmed to a diameter of 10 mm and a thickness of 450 µm. Finally, sterilization was performed using Co^60^ radiation.

### Histological examination of materials

APCS and human donor corneas were processed for routine paraffin embedding. Briefly, 5-µm-thick sections were cut and stained with hematoxylin and eosin (H&E). Tissues were also cut into pieces and processed for scanning electron microscopy (SEM, Hitachi SU8100) examination as described previously [14]. SEM images were obtained at a total magnification of 1000×.

### Surgical technique

Deep anterior lamellar keratoplasty (DALK) surgery was performed in a minimally modified manner as previously described [4]. Briefly, after trephination of approximately 2/3 of the total host corneal thickness using a Hessburg-Barron vacuum trephine, the remaining stroma was removed to a level close to the DM using a manual technique. Baring of DM was achieved in two cases of descemetocele. The recipient bed was 1 mm larger than the infiltration size. Additionally, in cases with bacterial and fungal keratitis, the exposed recipient bed was intraoperatively irrigated with antibacterial (ceftazidime) or antifungal (fluconazole) agents. Anterior chamber irrigation was performed in cases with sterile hypopyon. Since the APCS was dehydrated for preservation, it needed to be soaked in saline for 1 min before it was used for trephination in keratoplasty. APCS graft buttons were oversized by 0.25 mm and fixed to the recipient bed with interrupted 10-0 nylon sutures (Alcon, Fort Worth, TX).

### Postoperative treatment

To prevent recurrent infection, we postoperatively administrated multiple topical and systemic antimicrobial medications. For nonviral keratitis, systemic antimicrobial treatments [cephalosporin (ceftazidime) or fluoroquinolones (levofloxacin) for bacterial keratitis, according to the causative microbe and drug-susceptibility and voriconazole for fungal keratitis] were administered within 1 to 2 weeks postoperatively, while topical antimicrobial medications were usually maintained for at least 1 month after surgery and then tapered over the ensuing weeks if no infection recurred. For HSK, the postoperative antiviral treatment regimen comprised oral acyclovir 400 mg twice a day for 12 months and topical antiviral medications (ganciclovir and interferon) for at least 3 months. In cases of fungal and *Acanthamoeba* keratitis, topical steroids were withheld for at least 2 weeks, whereas for bacterial and viral infections, topical steroid treatment was initiated immediately after surgery. Topical steroids were generally prescribed four times daily for 6 months, gradually tapered down to a maintenance dose of once daily over a 3-month period, and then continued for another 3 months. Topical tacrolimus was used right after the surgery as an additional immunosuppressive medication and for its anti-inflammatory effect. Sutures were removed approximately 3 months after surgery.

### Clinical evaluation

Clinical measurements included visual acuity, graft survival and postoperative complications. The size of the corneal lesion was reported as the equivalent circular diameter (ECD) and was calculated as previously described [15]. Corneal graft failure was defined as the irreversible loss of central graft clarity. A diagnosis of recurrent infection was made according to the constellation of characteristic clinical manifestations, such as significant epitheliopathy, new corneal infiltration, positive confocal microscopy, or positive microbial culture. Recurrent infection after lamellar keratoplasty usually started from the graft-host junction or the stromal bed. Graft rejection was diagnosed on the basis of decreased vision and stromal edema with or without anterior segment inflammation. If the cause of graft failure was considered multifactorial, the precipitating event that eventually resulted in graft failure was chosen.

### Statistical analysis

Potential risk factors, including indications for grafting, the lesion size, the graft size and preoperative hypopyon, were assessed for associations with the incidence of graft failure. The statistical analyses were performed using SPSS 23.0 (SPSS Inc., Chicago, Illinois, USA) or R 3.6.0 (https://www.R-project.org/). Comparisons of continuous data among groups were performed by applying Student’s *t*-test. Graft survival was evaluated by the Kaplan–Meier method, and comparisons were performed using the log-rank test. Due to the presence of nonproportional hazards between the nonviral and herpetic keratitis groups, the weighted log-rank test was used to compare the survival curves. A weighted log-rank test was carried out using the comp() function in the R package “survMisc”, and Fleming–Harrington weights of P = 0, and q = 0.5 were chosen to emphasize differences later in time.

## Results

### Patient characteristics

**Fig. 1:**
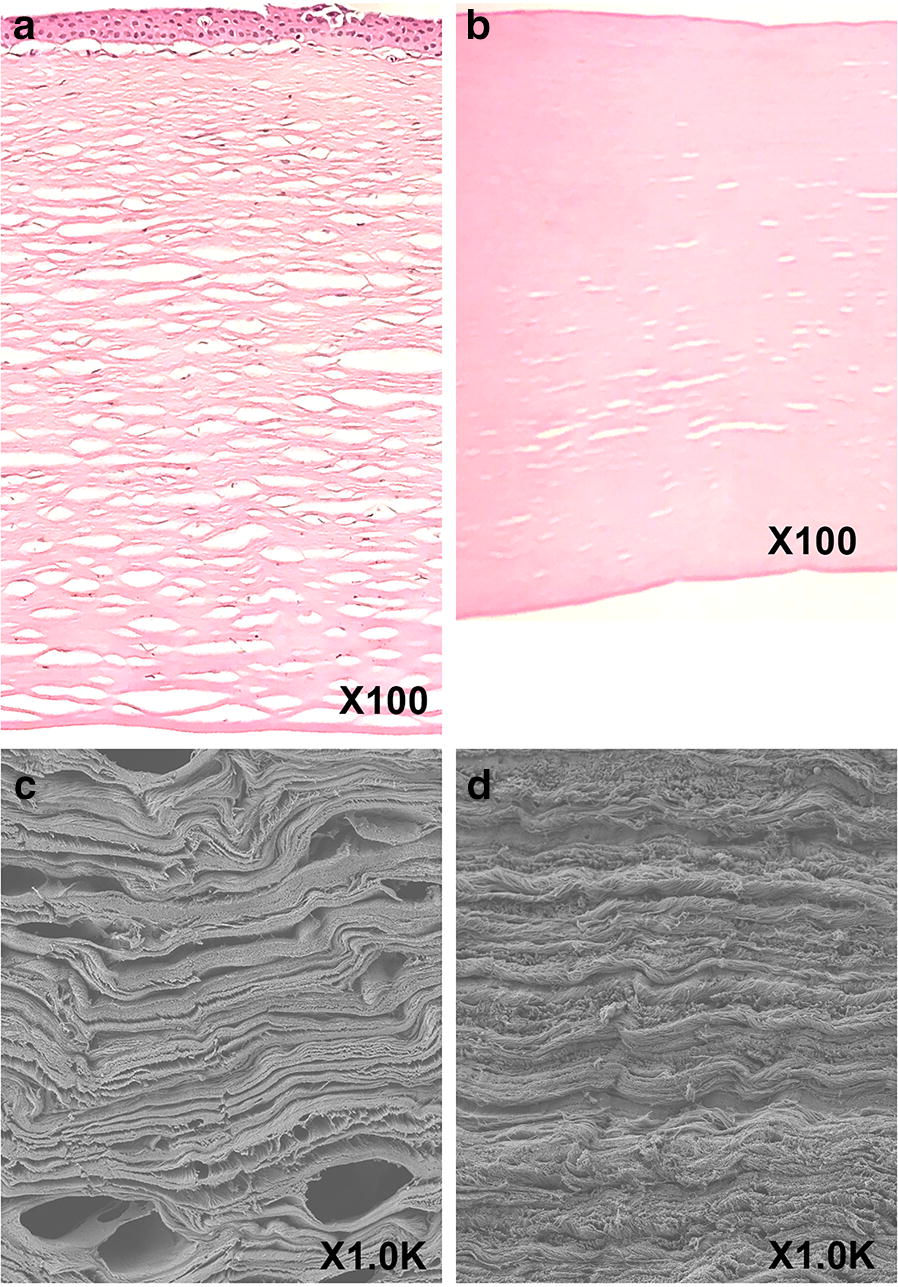
Architecture of acellular porcine corneal stroma (APCS). Hematoxylin and eosin (H&E) staining of APCS (**a**) and human cornea (**b**). Total magnification = 100×. Scanning electron micrography (SEM) images of APCS (**c**) and human cornea (**d**). Total magnification = 1000×

Thirty-nine eligible patients (39 eyes) were implanted with APCS to treat infectious keratitis. As shown in H&E histological stains, these replacements consisted of highly organized collagen fibers, yet no remaining cell (nuclei) were detected. SME revealed that the APCS reserved the structure of a native human corneal stroma but was less porous than the human cornea (Fig. 1).

Demographic features of the recipient are presented in Table 1. There were 14 females and 25 males with a mean age of 48.5 ± 13.9 years (range, 14–72 years). Fungal keratitis was the most common indication for surgery (n = 22, 56.4%), and was followed by HSK (n = 7, 17.9%), bacterial keratitis (n = 5, 12.8%) and *Acanthamoeba* keratitis (n = 4, 10.3%). One case was diagnosed as fungal keratitis with concomitant bacterial infection. The size of the corneal lesions ranged from 2.78 to 7.34 mm (mean, 5.15 ± 1.23 mm). Descemetocele formation was observed in two patients with HSK (5.1%). Sterile hypopyon due to intraocular inflammation rather than active intraocular infections was noted in 4 cases (10.3%). Two of these cases were diagnosed with fungal keratitis, one case with *Acanthamoeba* keratitis and once case with fungal keratitis with concomitant bacterial infection. The trephination sizes of the recipients ranged from 7.25 to 8.50 mm (mean, 7.70 ± 0.32 mm).

**Table 1:** Demographic and clinical characteristics

	Mean ± SD (range) or n (%)
Age (years)	45.5 ± 13.9 (14–72)
Lesion size^a^ (mm)	5.15 ± 1.23 (2.78–7.34)
Pathogens (n = 39)	
Fungal keratitis	22 (56.4%)
HSK	7 (17.9%)
Bacterial keratitis	5 (12.8%)
*Acanthamoeba* keratitis	4 (10.3%)
Combined fungal/bacterial keratitis	1 (2.6%)
Descemetocele	2 (5.1%)
Sterile hypopyon	4 (10.3%)

*SD* standard deviation, *n* No. of eyes, *HSK* herpes simplex keratitis, *ECD* equivalent circular diameter

^a^Lesion size is expressed as the equivalent circular diameter

### Visual acuity

**Fig. 2:**
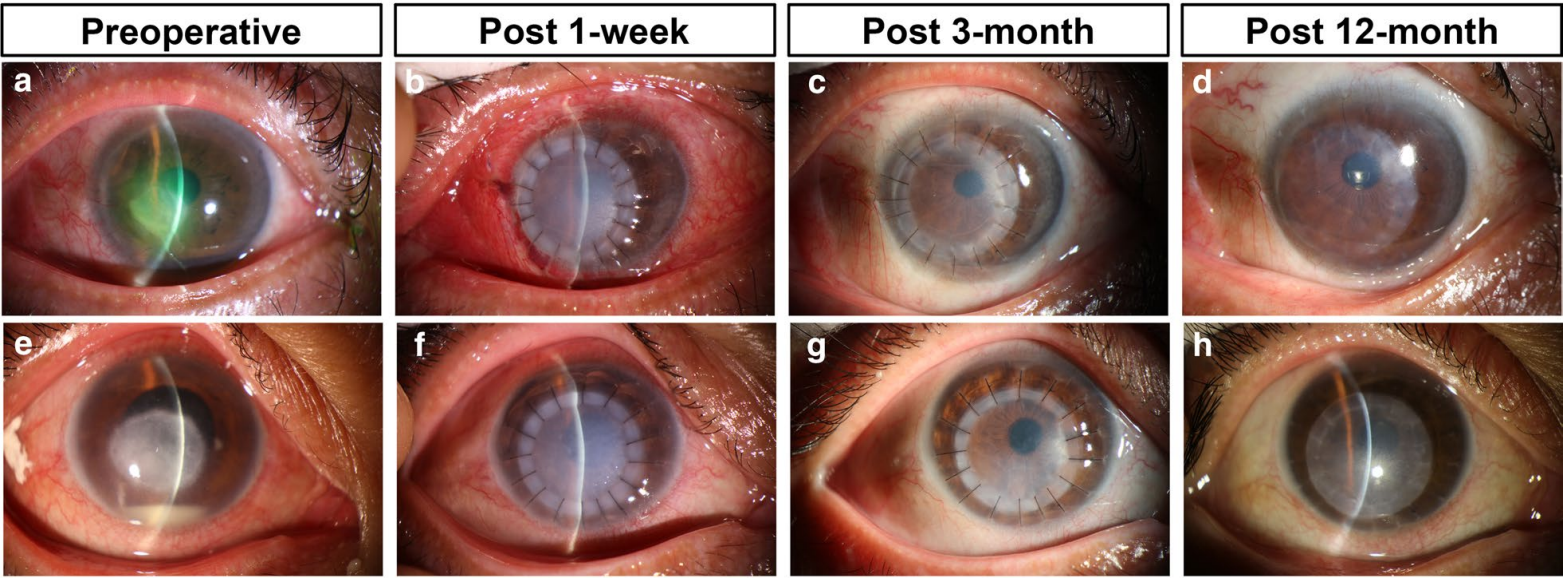
Pre- and postoperative outcomes of 3 patients who underwent therapeutic lamellar keratoplasty using acellular porcine corneal stroma (APCS) for medically unresponsive infectious keratitis. Images were obtained in eyes before surgery (**a**, **e**), and at 1 day (**b**, **f**), 3 months (**c**, **g**) and 12 months (**d**, **h**) postoperatively

**Fig. 3:**
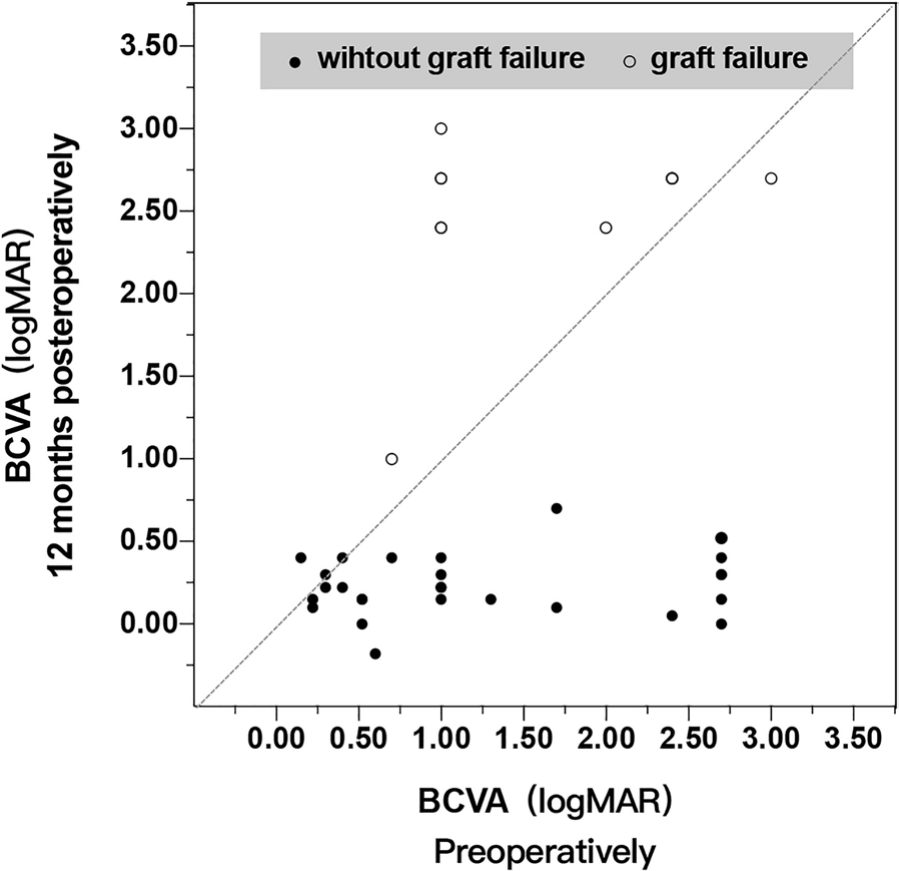
Visual outcomes in eyes undergoing acellular porcine corneal stroma (APCS) transplantation. The majority of patients had improved best-corrected visual acuity (BCVA) at 12 months after surgery. The points on the diagonal line show that the BCVA was the same preoperatively and 12 months postoperatively. Counting fingers, hand motion and light perception are shown as logarithm of the minimum angle of resolution (logMAR) values of 0.004, 0.002 and 0.001, respectively

Corneal transparency was restored following APCS transplantation in cases without graft failure (Fig. 2). Before keratoplasty, the mean best corrected visual acuity (BCVA) was 1.38 ± 0.94 logMAR, and only 10.3% (n = 4) of the eyes had a visual acuity of 20/40 or better. At the 12-month postoperative examinations, 51.2% (n = 20) of the treated eyes had a visual acuity of 20/40 or better, and in 85.2% (23/27) of the eyes that did not present graft failure, visual acuity increased by two or more Snellen lines (Fig. 3). A few of the patients were followed up for up to 3 years. They showed good preservation of visual acuity and transparent APCS grafts (Additional file 1: Figure S1).

### Graft survival and risk factors

**Fig. 4:**
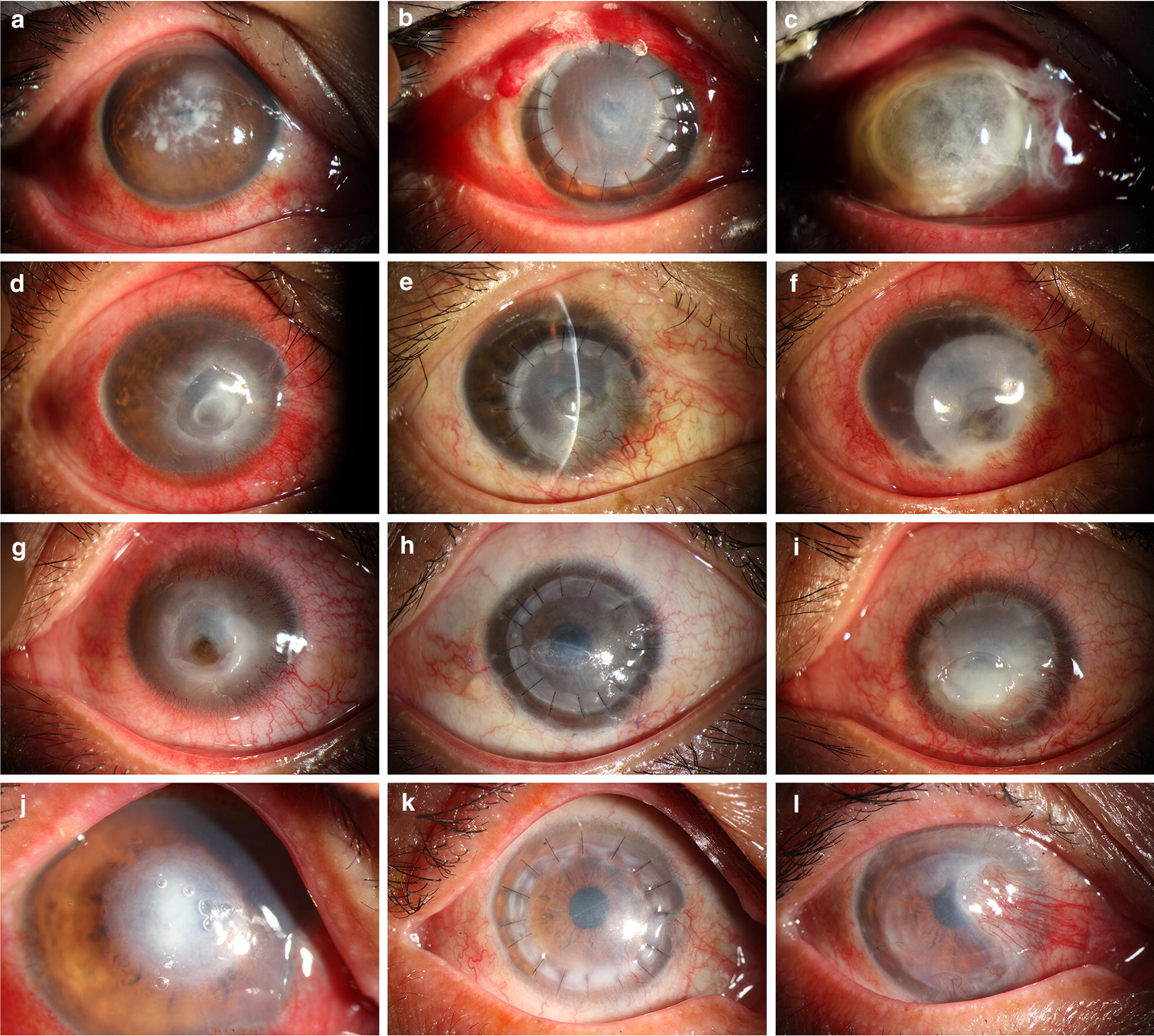
Slit-lamp microscopy images of eyes with graft failure. **a**–**c** A patient was diagnosed with fungal keratitis who experienced recurrent infection within 1 week after the operation. A repeated PKP was performed. **d**–**f** A patient with mild eyelid deformity and lagophthalmos, which resulted in neovessel ingrowth and led to late graft dissolution. **g**–**i** A patient with a diagnosis of herpes simplex keratitis who had persistent epithelial defects after the operation that ultimately progressed to corneal ulceration. **j**–**l** The graft was transparent in the initial postoperative period, but neovessels later grew into the center of the graft

Twelve patients presented graft failure within 1 year after keratoplasty (Fig. 4 and Table 2). Of the eyes with graft failure, two underwent repeat PKP and the remaining eyes were treated medically. Noninfectious graft melting was the major cause of graft failure (5/12, 41.7%); the pathogeneses included extensive inflammation (n = 3), neovessel ingrowth (n = 1) and persistent epithelial defects (n = 1). The other causes of graft failure were recurrence of primary infection (4/12, 33.3%) and extensive graft neovascularization (3/12, 25%). Recurrent fungal infection occurred within 1 month after surgery, and two of these cases occurred within the first postoperative week. The time to recurrence in HSK cases was approximately 8 months. No graft rejection occurred during the follow-up period.

**Table 2:** Clinical characteristics of patients with graft failure

Cause of graft failure	Indications	Lesion size^b^ (mm)	Graft size (mm)	Graft survival time (months)	Prognosis
Infection recurrence					
1	Fungal	3.39	7.50	0.2	Repeated PK
2	Fungal	6.01	8.50	0.1	Repeated PK
3	Fungal	6.51	7.75	0.5	Controlled
4	HSK	5.80	8.00	8.0	Controlled
Graft melting^a^					
5^c^	HSK	7.18	8.00	2.2	Controlled
6^d^	HSK	5.36	7.75	3.0	Controlled
7^d^	HSK	7.16	8.25	1.0	Controlled
8^d^	*Acanthamoeba*	5.60	8.25	4.3	Repeated PK
9^e^	Fungal	5.89	7.75	5.0	Repeated PK
Extensive graft neovascularization					
10	Fungal	6.22	8.00	6.5	Controlled
11	Fungal	5.42	8.50	6.5	Controlled
12	Bacterial	6.52	7.25	6.0	Controlled

*HSK* herpetic simplex viral keratitis, *PK* penetration keratoplasty

^a^Noninfectious graft melting

^b^Lesion size reported as the equivalent circular diameter

^c^Graft melting caused by persistent epithelial defect

^d^Graft melting caused by extensive inflammation

^e^Graft melting caused by neovessel ingrowth

**Fig. 5:**
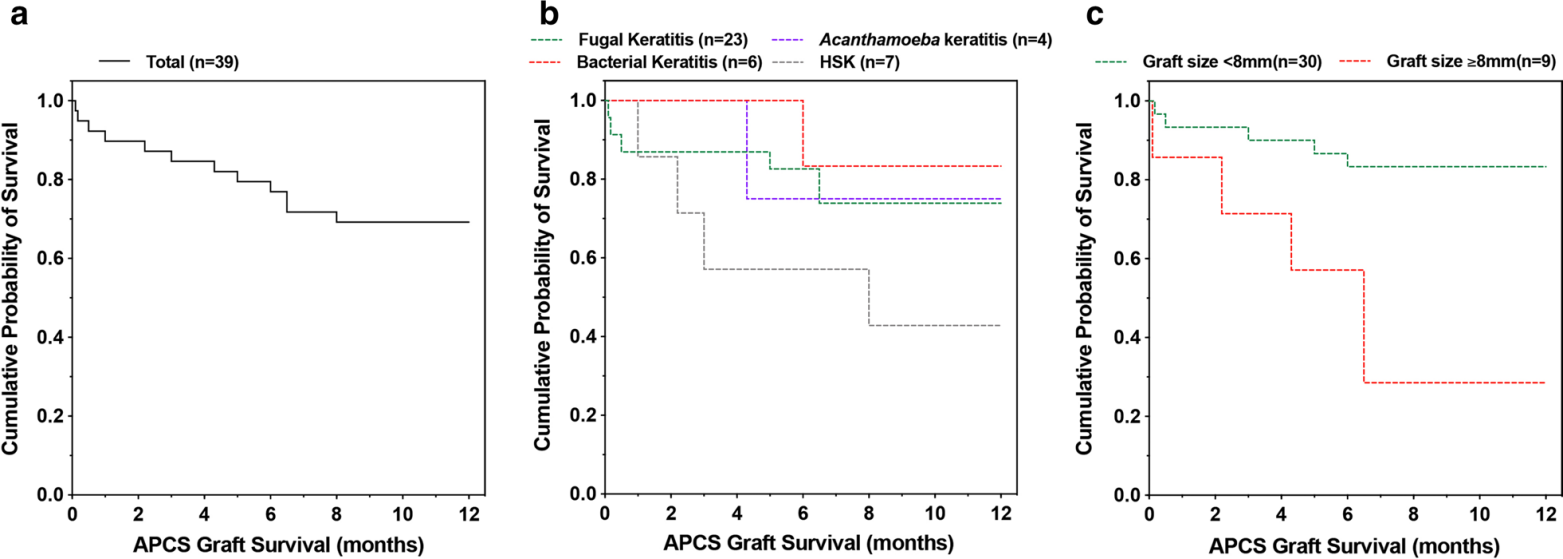
Kaplan–Meier survival curve depicting the graft failure episodes in eyes submitted to acellular porcine corneal stroma (APCS) implantation. **a** Total graft survival; **b** graft survival in eyes with various types of infectious keratitis; **c** graft survival in eyes with a graft size larger or smaller than a diameter of 8 mm. HSK, herpes simplex keratitis

The preoperative factors that influenced graft outcome are shown in Fig. 5 and Table 3. The risk was markedly higher in eyes with HSK than in eyes with nonviral keratitis [relative risk (RR) = 8.0, P = 0.046]. The use of grafts with a diameter equal or larger than 8.0 mm was also associated with a significantly higher risk of graft failure (RR = 6.5, P < 0.001). Eyes that developed graft failure had a slightly larger corneal lesion size than eyes that achieved therapeutic success (5.92 ± 1.00 mm versus 4.81 ± 1.17 mm, P = 0.007). Preoperative hypopyon did not present a high risk of graft failure (P = 0.198).

**Table 3:** Possible risk factors for graft failure

Parameter	Therapeutic success (n = 27)	Graft failure (n = 12)	P	Type of statistical test
Indication for graft			0.046	Weighted log-rank test^b^
Nonviral	24 (75.0%)	8 (25.0%)		
Herpetic	3 (42.9%)	4 (57.1%)		
Mean lesion size^a^ (mm)	4.81 ± 1.17 (2.78–7.34)	5.92 ± 1.00 (3.39–7.18)	0.007	*t*-test
Mean graft size (mm)	7.59 ± 0.21 (7.25–8.00)	7.95 ± 0.38 (7.25–8.5)	< 0.001	*t*-test
Graft size group			< 0.001	Log-rank test
< 8.0	25 (83.3%)	5 (16.7%)		
≥ 8.0	2 (22.2%)	7 (77.8%)		
Preoperative sterile hypopyon			0.198	Log-rank test
Absence	23 (65.7%)	12 (34.3%)		
Presence	4 (100%)	0 (0%)		

^a^Lesion size reported as the equivalent circular diameter

^b^Because of the presence of nonproportional hazards, the weighted log-rank test was used to compare the survival distributions between nonviral keratitis and herpetic keratitis groups. Fleming–Harrington weights of P = 0, q = 0.5 were set to emphasize differences occurring later in time

## Discussion

Corneal allotransplantation is a well-established technique for treating corneal blindness. However, there is a worldwide shortage of human donor corneas, and this has led to a demand for the development of biocompatible materials as an alternative [16]. Enormous progress has been made in this field, and in recent years APCS has moved into the clinical evaluation stage [10, 17–19]. A main advantage of APCS over human donor corneas is its unlimited availability. However, APCS has only been reported for therapeutic indications of keratoplasty in fungal and herpetic keratitis [10, 11]. The current study further supports the viability of APCS as an alternative to the human cornea in cases of progressive infectious keratitis caused by a wide variety of pathogens, such as viruses, fungi, bacteria, and *Acanthamoeba*. This study is also the first to disclose the major risk factors for APCS graft failure, and this information may help in future patient selection and the postoperative management of APCS transplantation.

Corneal transplantation performed for active infectious keratitis is associated with a high risk of failure [20]. The graft survival rate varied depending on the treatment conditions [5]. For instance, in a study by Aushu et al. the therapeutic success rate of lamellar keratoplasty performed using a human cornea was 86.6% in nonviral infectious keratitis [4], and Zhang et al. reported that APCS transplantation achieved therapeutic success in 87.2% (41/47) of fungal keratitis cases [10], indicating that the rate of success was comparable between human donor corneas and APCS. In our study, the therapeutic success rate of APCS transplantation in nonviral infectious keratitis was 75% (24/32), and this rate was a slightly lower than that reported by Zhang et al. The severity of corneal infection might contribute to the differences observed between these two studies. In addition, visual rehabilitation is a secondary consideration in therapeutic keratoplasty. Both previous studies and this study showed that APCS transplantation can restore the optical clarity of the cornea, and the majority of recipients without graft failure eventually had improved visual acuity [10]. Therefore, all of these results collectively suggest that APCS meets the clinical demands for xenografts in various types of infectious keratitis.

Graft rejection is always the primary concern in xenotransplantation. While APCS implantation is by its nature a xenotransplantation, no evidence of graft rejection was observed in any of our patients at the last follow-up. Our findings are consistent with those presented in previous reports [10, 21]. Fresh porcine corneal stroma contributes only 1.62% and 6.12%, respectively, of the cellular and humoral immunogenicity of an intact cornea [22]. During the process of tissue decellularization, cellular material and antigen molecules are nearly completely removed, significantly alleviating the host immune response following its transplantation [21]. A previous study showed that transplantation of APCS into corneal pockets and subcutaneous tissue was incapable of triggering an accepted immune response [13]. Indeed, a significant advantage of APCS is its inherently low antigenicity and immunogenicity. However, it is worth noting that collagen retains weak antigenic determinants in its telopeptide regions. Moreover, APCS contains, in addition to collagen, a very few small number of antigenic noncollagenous proteins and residual cell-associated components [23]. In this regard, although no host-versus-graft type rejection was observed in this or previous studies, anti-rejection therapy should be routinely postoperatively administered to APCS-implanted eyes. Nevertheless, the duration of anti-rejection medication requires further investigation.

In our study, five patients developed noninfectious graft melting, which is the most common cause of graft failure. Graft melting is a devastating complication of corneal transplantation. It is a destructive process involving collagen dissolution driven by the excessive release of tissue collagenase [24]. One group of these collagenases is the matrix metalloproteinases (MMPs) [25]. Graft melting occurs under a number of conditions, including infectious or sterile inflammation. Under sterile conditions, such as intense inflammation, persistent corneal epithelial defect and corneal neovascularization, proinflammatory cytokines are released, resulting in increased collagenases activity and subsequent graft melting [26–28]. Collagen-based corneal stroma substitutes are generally more prone than human donor corneas to collagenase dissolution [29]. However, APCS preserves the interwoven lamellar structure of the natural cornea, and this endows it with better resistance to collagenase than is achieved by the biosynthetic collagen materials. Therefore, graft melting does not seem to be an overly concerning issue with regard for use of APCS.

Several preoperative factors that exerted a profound impact on graft survival were identified in this study. First, we noted that APCS transplantation had a much worse prognosis in clinically active herpetic eyes than in eyes with nonviral microbial keratitis. In our series, the 1-year graft survival rate for cases of bacterial, fungal and *Acanthamoeba* keratitis varied from 73.9 to 85.7%; in contrast, only 42.9% (3/7) of our patients with HSK achieved therapeutic success. The extremely low survival rate of APCS grafts in active herpetic keratitis observed in this study is consistent with previous reports. For example, Zhang et al. performed APCS transplantation to treat 13 cases of herpes simplex virus (HSV)-related corneal ulcers or scarring [11] and observed graft failure in 15% of quiescent eyes but up to 71.43% (5/7) of inflamed eyes during the follow-up period. Therefore, similar to human donor cornea, APCS transplantation is not recommended in patients with clinically active HSK.

However, under some conditions, such as medically uncontrolled ulceration, ulceration at risk of perforation, and insufficient fresh donor corneas to meet demands, APCS transplantation may be applied. There are some management considerations that could improve surgical prognosis. HSK is typically characterized by recurrent episodes of infections, because HSV can be latent in the trigeminal ganglion [30]. After keratoplasty, HSV is able to migrate into the graft along with the regenerated subbasal nerve fibers [11, 31]. HSK patients may therefore remain at risk of recurrent infection beyond the initial postoperative period. Sufficient antiviral courses are therefore necessary in these patients. Moreover, in the present study, we found that noninfectious graft melting was the most common cause of graft failure in HSK patients. Possible recognized predisposing factors for APCS graft melting in HSK patients include ongoing ocular surface inflammation and a persistent epithelial defect. A previous study revealed that early postoperative persistent epithelial defects were present in 8% of HSK cases that underwent PK and 44% of cases that underwent LK [32]. These inflammatory settings could result in elevated levels of collagenases such as MMP, being produced by inflammatory cells, predominantly including neutrophils [26–28]. Therefore, in this regard, the prevention of corneal epithelial defects and efficient anti-inflammatory medication are particularly necessary to achieve therapeutic success in eyes undergoing APCS transplantation for active HSK.

In our study, another preoperative factor associated with the incidence of graft failure was graft size. We found that APCS grafts performed with a size ≥ 8.0 mm had a higher risk than smaller grafts of developing recurrent infection (22.2% vs 6.7%), graft melting (33.3% vs 6.7%) or extensive graft neovascularization (22.2% vs 3.3%). Williams et al. and Volker-Dieben et al. conducted a large retrospective analysis to evaluate the effect of graft size on graft outcomes. Both of these authors showed that graft failure rates were higher for grafts exceeding of 8.0 mm [33, 34]. In therapeutic PKP for infectious keratitis, Killingsworth also reported that graft size significantly influenced therapeutic success and that survival was better for smaller grafts (< 9.0 mm) than larger grafts (≥ 9.0 mm) in bacterial (83% vs 50%) and fungal (75% vs 33%) keratitis [35]. The original size of the APCS grafts used in this study was 10 mm, the APCS grafts were trephined into the size of choice during keratoplasty. In our study, trephines of 7.25 to 8.25 mm in diameter were used, and these sizes were larger than that those used in the recently published study by Zhang and colleagues (6.25–7.50 mm) [11]. We found that APCS grafts performed with a size ≥ 8.0 mm resulted in a much worse survival rate than was achieved in for smaller grafts (83.33% vs 11.11%, RR = 6.5). Two explanations might be considered for this findings: (a) the use of a large graft might increase the chance of an immune response derived from the corneal limbus; and (b) a larger graft size might also indicate the presence of more several preoperative infections [36].

Moreover, we found that compared to human corneal transplantation, in APCS transplantation, the graft size seemed to exert a more significant influence on graft failure. We suggest that this difference could be due to two reasons. First, as we discussed above, the use of a large graft might increase the chance that the graft will interact with immune cells derived from the corneal limbus. Even worse, APCS grafts are more prone than human donor corneas to interact with collagenase released by immune cells than donor human cornea [29]. Second, because it is a xenotransplantation product, the residual antigenicity of APCS should not be overlooked [23]. If a graft with a larger size is used, APCS grafts might have a higher risk of presenting antigens to limbal immune cells and thereby inducing an immune response. Therefore, according to our findings, the use of APCS with a diameter larger than 8.0 mm should potentially be avoided.

A relatively short follow up period is a limitation of this study. Because serious complications, such as recurrent infectious and graft rejection, are more likely to occur within 1 year after corneal transplantation, our patients were followed up for at least 12 months. A future study will be designed to verify the long-term success of APCS transplantation for infectious keratitis.

## Conclusion

Based on these findings, although the number of patients was limited and the follow-up duration was short, this study provides some insights that will be valuable for the future application of APCS, including surgical indications and postoperative management.

## Supplementary information

**Additional file 1: Figure S1.** Slit-lamp biomicroscopy performed at 2 (**a**) and 3 (**b**) years after implantation with acellular porcine corneal stroma (APCS). The implant was well integrated into the recipient cornea. The patient had a visual acuity of 20/25 in the operated eye.

## Data Availability

All data generated or analyzed during this study are included in this published article.

## Abbreviations

PKP: penetrating keratoplasty
ALK: anterior lamellar keratoplasty
APCS: acellular porcine corneal stroma
DM: Descemet’s membrane
HSK: herpes simplex keratitis
PBS: phosphate-buffered saline
H&E: hematoxylin and eosin
SEM: scanning electron microscopy
DALK: deep anterior lamellar keratoplasty
ECD: equivalent circular diameter
BCVA: best corrected visual acuity
MMP: matrix metalloproteinase
HSV: herpes simplex virus
