# Supplementary figures and images for: Risk factors influencing survival of acellular porcine corneal stroma in infectious keratitis: a prospective clinical study
Source: J Transl Med. 2019 Dec 30;17:434. doi: 10.1186/s12967-019-02192-z (PMC6941327; doi:10.1186/s12967-019-02192-z)

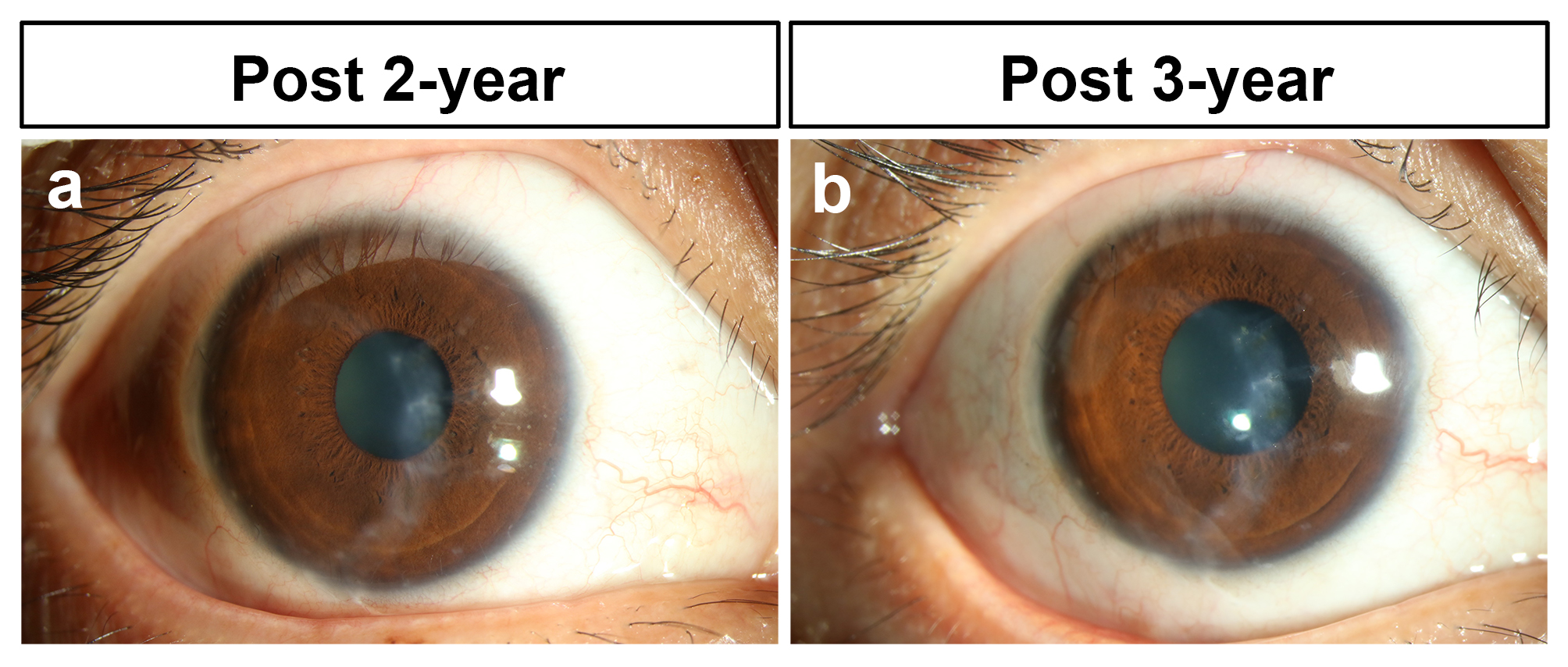

Supplement: Supplementary file 1 — Additional file 1: Figure S1. Slit-lamp biomicroscopy performed at 2 (a) and 3 (b) years after implantation with acellular porcine corneal stroma (APCS). The implant was well integrated into the recipient cornea. The patient had a visual acuity of 20/25 in the operated eye. [file 12967_2019_2192_MOESM1_ESM.jpg]
